# Intestinal Permeability and Circulating CD161+CCR6+CD8+T Cells in Patients With Relapsing–Remitting Multiple Sclerosis Treated With Dimethylfumarate

**DOI:** 10.3389/fneur.2021.683398

**Published:** 2021-08-26

**Authors:** Maria C. Buscarinu, Francesca Gargano, Luana Lionetto, Matilde Capi, Emanuele Morena, Arianna Fornasiero, Roberta Reniè, Anna C. Landi, Giulia Pellicciari, Carmela Romano, Rosella Mechelli, Silvia Romano, Giovanna Borsellino, Luca Battistini, Maurizio Simmaco, Corrado Fagnani, Marco Salvetti, Giovanni Ristori

**Affiliations:** ^1^Department of Neuroscience, Mental Health and Sensory Organs, Sapienza University, Rome, Italy; ^2^Neuroimmunology Unit, Istituto di Ricovero e Cura a Carattere Scientifico (IRCCS) Fondazione Santa Lucia, Rome, Italy; ^3^Mass Spectrometry Section, Laboratory of Clinical Biochemistry Sant'Andrea University Hospital, Rome, Italy; ^4^Department of Clinical-Experimental Neuroscience and Psychiatry, Sapienza University, Rome, Italy; ^5^Department of Human Science and Promotion of Quality of Life, San Raffaele Roma Open University, Rome, Italy; ^6^Center for Behavioral Sciences and Mental Health, Higher Institute of Health (ISS), Rome, Italy; ^7^Istituto di Ricovero e Cura a Carattere Scientifico (IRCCS) Istituto Neurologico Mediterraneo (INM) Neuromed, Pozzilli, Italy

**Keywords:** multiple sclerosis, intestinal permeability, CD161+CCR6+CD8+T cells, mucosal immunity, dimethyl-fumarate

## Abstract

**Background:** The changes of the gut-brain axis have been recently recognized as important components in multiple sclerosis (MS) pathogenesis.

**Objectives:** To evaluate the effects of DMF on intestinal barrier permeability and mucosal immune responses.

**Methods:** We investigated intestinal permeability (IP) and circulating CD161+CCR6+CD8+T cells in 25 patients with MS, who met eligibility criteria for dimethyl-fumarate (DMF) treatment. These data, together with clinical/MRI parameters, were studied at three time-points: baseline (before therapy), after one (T1) and 9 months (T2) of treatment.

**Results:** At baseline 16 patients (64%) showed altered IP, while 14 cases (56%) showed active MRI. During DMF therapy we found the expected decrease of disease activity at MRI compared to T0 (6/25 at T1, *p* = 0.035 and 3/25 at T2, *p* < 0.00), and a reduction in the percentage of CD161+CCR6+CD8+ T cells (16/23 at T2; *p* < 0.001). The effects of DMF on gut barrier alterations was variable, without a clear longitudinal pattern, while we found significant relationships between IP changes and drop of MRI activity (*p* = 0.04) and circulating CD161+CCr6+CD8+ T cells (*p* = 0.023).

**Conclusions:** The gut barrier is frequently altered in MS, and the CD161+ CCR6+CD8+ T cell-subset shows dynamics which correlate with disease course and therapy.

## Introduction

Multiple sclerosis (MS) is a chronic disease of the central nervous system (CNS), with inflammation, demyelination, and neurodegeneration. The pathogenic process is immune-mediated, and the etiology is probably multifactorial, with interaction of heritable and non-heritable factors (1).

Among the other factors, microbiota and gut function are increasingly recognized as relevant in this immune-mediated disorder (2). Several studies have recently shown that the microbiota, as a part of the intestine–brain axis, plays a role in the etiopathogenesis of MS (3, 4). However, a crucial component of this axis, the intestinal barrier, has received much less attention. The question of whether or not intestinal permeability (IP) is affected during the disease course is at least as important as the changes in the microbiota balance (5, 6).

IP changes may underlie gastro-intestinal or even far-from-gut autoimmune disorders. In fact, increased gut permeability allows the passage of macromolecules, toxins, and bacterial species that may trigger immune-mediated diseases in different systems, even distant from the gastrointestinal tract, such as the CNS (7, 8). On the other hand, CNS inflammation can increase gut permeability and alter mucosal structure in the small intestine (9).

In a previous work, we investigated the gut permeability in relapsing–remitting MS (RRMS) patients and healthy donors, finding that alteration of IP represents a relatively frequent event in patients with MS (10). This study and a previous one, showing that CD161^high^CD8+ T cells, encompassing the mucosal associated invariant T (MAIT) cell subset, play a role in MS pathogenesis (11), prompted us to focus on the gut triggers that may lower the threshold for disease development in susceptible individuals.

Dimethylfumarate (DMF) has both neuroprotective and anti-inflammatory effects, and it is currently used as an oral, first-line, disease-modifying therapy (DMT) in MS. Some of the mechanisms responsible for its efficacy have been clarified, while others remain unexplored. Gastrointestinal tract irritation is one of the most frequent side effects of DMF (12). On the other hand, studies on experimental models of inflammatory bowel diseases showed that DMF might beneficially affect IP (13).

In this study, we investigated IP changes, the circulating CD161+CD8+ T-cell subset, and clinical/neuroradiological data in a cohort of RRMS patients before and after 9 months of DMF therapy, with a longitudinal design aimed at analyzing data at three time points: baseline (before therapy) and after 1 (T1) and 9 months (T2) of treatment.

## Methods

### Subjects and Procedures

Twenty-five patients, candidate to DMF therapy according to the approved indications, were enrolled and completed the follow-up. The other inclusion criteria were as follows: age between 18 and 60 years; a treatment-naïve status or being free from “first-line” DMT for at least 3 months; EDSS up to 5.5.

The exclusion criteria were the following: any serious internal medicine disease; any condition that may possibly interfere with the IP test, such as gastrointestinal disorders, renal function, and bladder dysfunction; pregnancy and breast-feeding. The study was conducted after approval of the local Ethics Committee, and a signed informed consent was obtained from each patient.

Each participant underwent the following procedures at baseline (T0) and after 1 (T1) and 9 months (T2) of DMF therapy, and in case of relapse: clinical evaluation, including the recording of gastrointestinal side effects after DMF start; data-sheet safety laboratory tests; urine sampling for IP test; blood sample for CD161+CD8+ T subset analysis; magnetic resonance imaging (MRI) of brain and spinal cord with gadolinium (Gd) to monitor the disease activity.

### MRI Protocol

All subjects underwent gadolinium (Gd)-enhanced MRI (brain and spinal cord). MRI was performed in all the patients with a 1.5-T magnet (Philips Gyroscan NT 1.5), with sequences Flair, T2- and T1-weighted after Gd. The presence of at least one Gd-enhancing lesion or of at least one new/enlarging T2-hyperintense lesion was considered indicative of disease activity at MRI.

### Intestinal Permeability Analysis

To evaluate IP, we used a solution composed of 5 g of lactulose and 2 g of mannitol in 50 ml of deionized water. All patients followed a lactulose-, mannitol-, lactose-free diet for 72 h before the test, as reported in a form delivered to the patient at the time of enrollment. After the assumption of the solution, the patients collected their own urine for the following 6 h, during which they have been encouraged to drink tap water. A pre-test urine sample was collected at the beginning and subtracted from the ending total. We calculated the total volume, and we stored 10 aliquots of 5 ml and 5 aliquots of 10 ml at −20°C until analysis. Lactulose and mannitol concentrations in urine samples were analyzed using a modified Liquid Chromatography Tandem Mass Spectrometry (LC-MS/MS) method (14).

The HPLC analysis was performed using an Agilent Liquid Chromatography System series 1100 (Agilent Technologies, USA). Chromatographic separation was performed using a column (Luna® Omega 3 μm SUGAR 100 Å, LC Column 100 × 2.1 mm, Ea Phenomenex, CA, USA) equipped with a security guard precolumn (Phenomenex, Torrance, CA, USA) containing the same packing material. The mobile phase consisted of a solution of HPLC-grade water (eluent A) and 100% HPLC-grade acetonitrile (eluent B); elution was performed at flow rate of 300 μl/min. The oven temperature was set at 40°C. The injection volume was 10 μl, and the total analysis time was 13 min. The mass spectrometry method was performed on a 3200 triple quadrupole system (Applied Biosystems, Foster City, CA, USA) equipped with a Turbo Ion Spray source. The detector was set in the negative ion mode. The Q1 and Q3 quadrupoles were tuned for the unit mass resolution. The transitions of the precursor ions to the product ions were monitored with a dwell time of 200 ms for each analyte. The instrument was set in the multiple reaction monitoring mode. Mass spectrometer parameters were optimized to maximize sensitivity for all analytes. Data were acquired and processed with Analyst 1.5.1 software. Therefore, we calculated the fractional excretion of lactulose as the following ratio, lactulose: lactulose (mg)^excreted^/lactulose (mg)^assumed^. We used the same method to evaluate excretion of mannitol. Our results have been reported as ratio of the lactulose fractional excretion to the mannitol fractional excretion (L/M ratio). Therefore, we were able to quantify the IP status: Lactulose:Mannitol *ratio* > 0.03 corresponded to an altered permeability, which had to be associated with a urinary mannitol concentration <900 mg/L (15).

### Flow Cytometry

Peripheral blood samples were collected into sodium heparin Vacutainer tubes (BD Biosciences, San Jose, CA) at baseline and at month 9 after starting DMF treatment. Peripheral blood mononuclear cells (PBMCs) were isolated from whole blood by density gradient centrifugation using standard procedures (Ficoll-Paque Plus, GE Healthcare). Fresh PBMC (*ex vivo*) from MS patients were labeled with antibodies directed to cell surface proteins along with a dead-cell discrimination reagent for 20 min at room temperature (RT) in the dark. The following antibodies were used: CD8 FITC (Biolegend), CCR6 Alexa Fluor 647 (Biolegend), CD3 BV605 (Becton Dickinson), CD4 BV785 (Becton Dickinson), CD161APC/Fire 750 (Biolegend), and Live/Dead Fixable Aqua Dead cell stain (Invitrogen) to define the frequency of CD8^+^ T cells (CD161^hi^, CCR6^+^). All antibodies were titrated to determine optimal concentrations. Stained cells were acquired on a CytoFLEX flow cytometer (Beckman Coulter), equipped with three lasers and able to measure up to 15 parameters simultaneously on each cell. For each sample, ~300,000 lymphocytes were selected based on scatter parameters, and the analysis was conducted after the exclusion of dead cells and coincident events. The data was compensated and analyzed using FlowJo v10.6.1 (TreeStar, Ashland, OR).

### Statistical Analysis

All variables were inspected for normal distribution. Between-group comparisons for continuous variables included parametric Student's *t*-test and ordinary one-way ANOVA, as well as non-parametric Kruskal–Wallis and Dunn multiple comparison tests (GraphPad Prism, v6.2). Between-group comparisons for categorical variables were performed by Pearson's chi-squared test. Statistical significance was inferred for *p*-values below 0.05.

Logistic regression models were fitted to describe the interplay between IP changes and the dynamics of CD161+CCR6+CD8+ T cells and MRI activity across the follow-up period. The same interplay was also explored by Kaplan–Meier and Cox proportional-hazard analysis based on time from MS onset. Multivariate and survival analyses were performed with the Stata software (version 16).

## Results

The demographic, clinical, and neuroradiological characteristics of 25 patients at baseline are summarized in Table 1. Sixteen patients (64%) showed an altered IP, while 14 cases (56%) showed active MRI (4 of them were also in clinical relapses). Moreover, we investigated the frequencies of CD161+ CCR6+ CD8+ T lymphocytes in PBMCs obtained from 23 MS patients.

**Table 1:** Demographic, clinical, and neuroradiological characteristics of patients at baseline.

Females/Males (*n*)	17/8
Age, years [mean (sd), range]	40.36 (12.41), 19–59
Disease duration, years [mean (sd), range]	7.28 (7.76), 1–32
EDSS [mean (sd), range]	1.64 (1.08), 0–5
Patients with relapse	4/25 (16%)
Patients with active MRI^*^	14 (56%)
DTM naive/free from DMT^**^	15/10

* *The presence of at least one Gd-enhancing lesion or of at least one new/enlarging T2-hyperintense lesion*.

** *Free from DMT for at least three months*.

**Figure 1:**
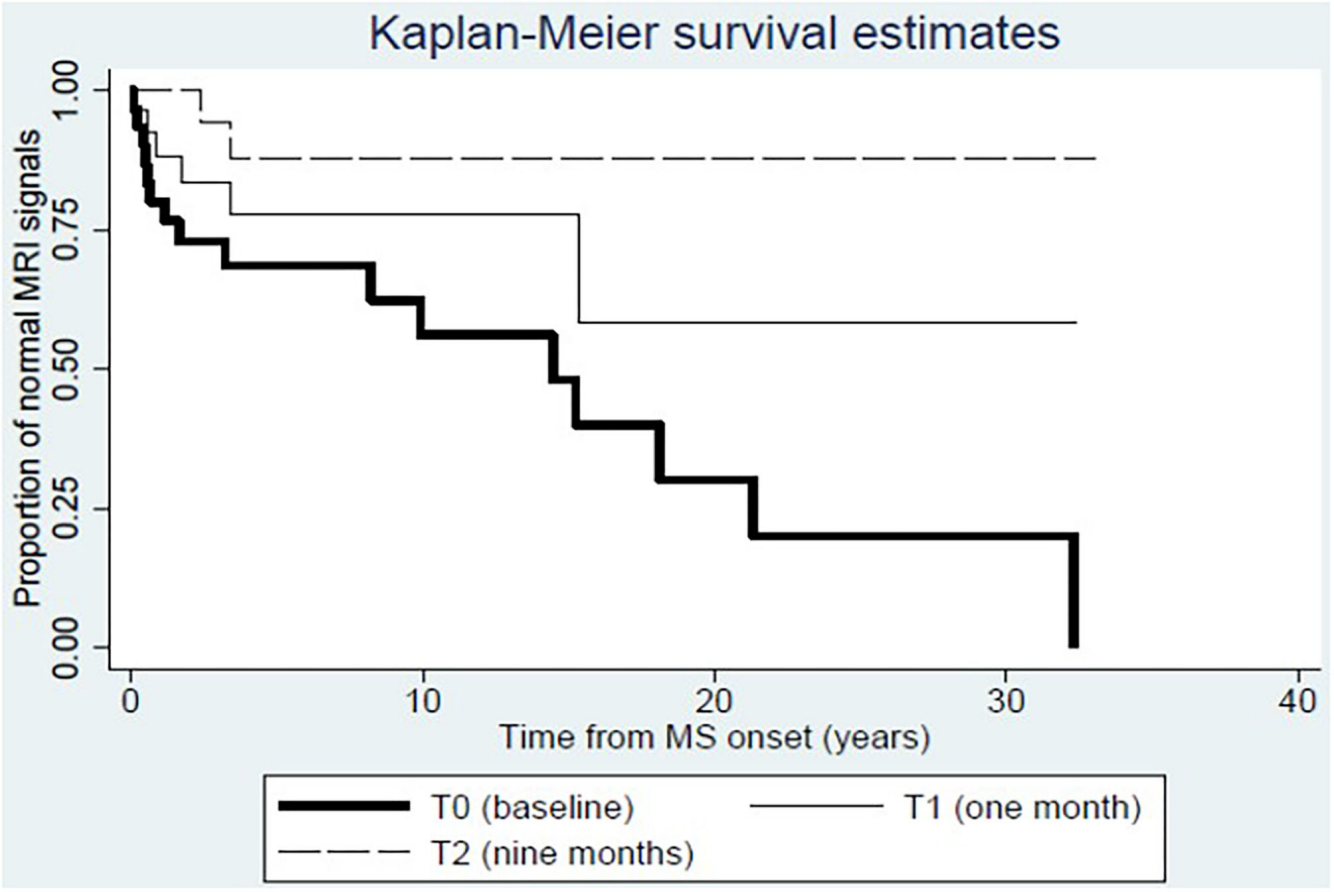
Dynamics of disease activity at MRI before and after dimethylfumarate therapy. Kaplan–Meier estimates show that the proportion of patients with normal MRI signal increases at T1 and T2 compared to T0.

**Figure 2:**
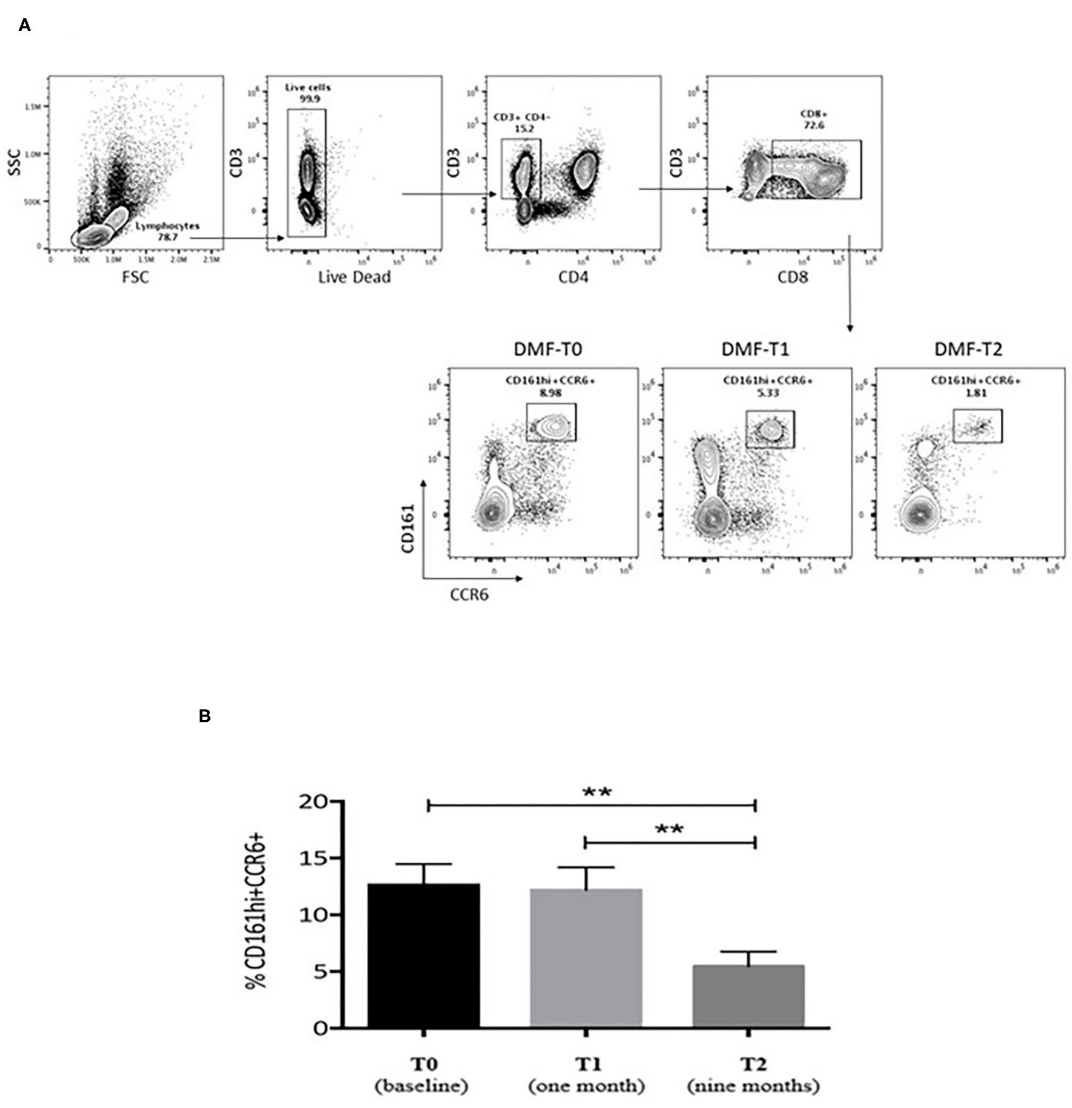
**(A)** Representative plots showing the frequencies of subpopulations of CD8 T cells (CD161hi CCR6+) in an MS patient during dimethylfumarate (DMF) treatment. Freshly isolated PBMCs were stained with CD3, CD8, CD4, CD161, and CCR6. For this study, we followed 23 patients undergoing treatment with DMF at different time points (3 and 9 months) after initiation of therapy to evaluate the *in vivo* impact of DMF on CD8 T cells in MS. Numbers indicate percent of cells in each quadrant. **(B)** Cumulative data for the frequencies of CD8+CD161hiCCR6+ T cells from the PBMCs of untreated MS patients at baseline (black bar, *n* = 23), T1 (1 month), and T2 (9 months of therapy; gray bars, *n* = 23). The T-cell subset dropped significantly at T2 compared to T0 and T1. Statistical comparisons were performed by ANOVA and Dunnett's multiple comparisons test. Significant values: ^**^p < 0.001.

During DMF therapy, two significant changes emerged. At first, we could confirm the decrease of disease activity as evaluated by MRI (6/25 at T1, *p* = 0.035 for the comparison between T1 and T0; 3/25 at T2, *p* < 0.001 for the comparison between T2 and T0); consistent with this result, the Kaplan–Meier analysis showed that the proportion of patients with normal MRI signal is higher at T1 and T2 compared to T0 (Figure 1). Then, we showed that the frequency of circulating CD161+CCR6+CD8+ T cells in MS patients is reduced after 9 months of DMF treatment. In Figure 2A, representative plots depict the progressive drop of frequencies of subpopulations of CD8 T cells (CD161hi CCR6+) in an MS patient during DMF treatment, while Figure 2B shows the cumulative data with the significant drop of the T-cell subset at T2 (*p* < 0.001).

**Figure 3:**
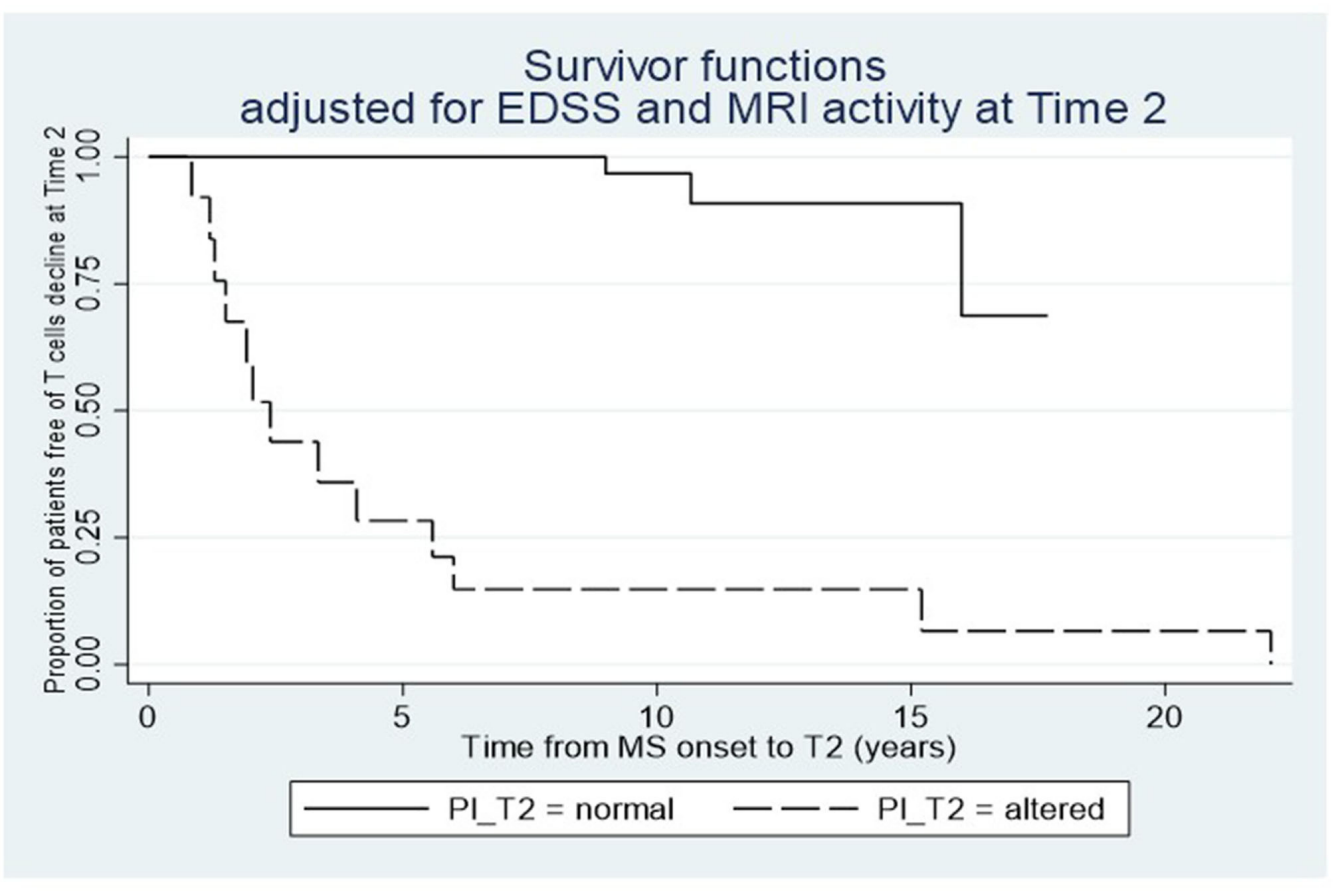
Relationship between the dynamics of circulating CD161+CD8+ T cells after dimethylfumarate therapy and the IP changes at T2. Cox proportional-hazard estimates show that the T-cell subset drop is higher in patients with IP alterations compared to those with normal IP at T2.

We found that the decrease in disease activity evaluated radiologically was 12 times higher in subjects showing reduced frequencies of CD161+CCR6+CD8+ T cells in the peripheral blood, and 15 times higher in cases with IP changes at T1 (Table 2). Furthermore, a logistic regression model showed a relationship between the drop of CD161+CD8+ T cells in the peripheral blood and IP changes at T2, considering as covariates both EDSS and MRI activity (*p* = 0.023). Consistent with this result, the Cox analysis showed that the decline of the T-cell subset was more evident in patients with persistent IP changes (Hazard Ratio, HR = 4.19; *p* = 0.03; Figure 3).

**Table 2:** Probability of MRI activity reduction at T2 in cases with parallel drop of CD161+CCR6+CD8+ T cells in blood, and in cases with IP changes at T1.

**Predictors**	**OR**	***P*-value**
**Logistic regression model for MRI activity drop at Time 2**		
**Drop of T cells at Time 2**		
No	1	
Yes	12.63	0.071
**IP change at T1**		
No	1	
Yes	15.42	0.040
Other covariates include EDSS and gastro-intestinal symptoms at baseline		

Concerning the possible effects of DMF treatment on gut barrier alterations, no significant difference emerged: some cases improved, while others worsened during follow-up, without a clear longitudinal pattern (Supplementary Table); the proportion of patients having IP changes at T1 and T2 was 16/25 (64%) and 15/25 (60%), respectively (figures that were quite comparable to the baseline data). A minority of patients (3/25) had mild gastro-intestinal side effects during treatment with DMF; other mild side effects were within the known safety profile of the drug (not shown). One patient presented a relapse during the follow-up, and no significant change occurred in patients' EDSS at T2 compared to baseline.

## Discussion

This work, together with our pilot studies on IP and mucosal immunity in RRMS (10, 11), provides evidences that the gut barrier is frequently altered in these patients and that the CD161+CD8+ T-cell subset shows dynamics compatible with disease course and therapy. No other studies on IP changes in MS have been reported so far, since the other works are mainly focused on microbiota changes (3). However, we deem IP alterations and dysbiosis as two faces of the same coin (16), and further studies correlating IP and gut microbiota changes in MS will certainly be informative on disease etiopathogenesis.

The CD161+CD8+ T-cell subset encompasses the MAIT cells, which were the object of several investigations after our first study on MS in 2011 (11), all largely confirming the involvement of MAIT cells in MS pathogenesis. Among the evidences repeatedly reported were an IL18-driven activation and consequent CNS infiltration of MAIT cells in the diseased brain, and an increased type-17 differentiation and oligoclonality of circulating MAIT cells in MS patients compared to controls (17–21). The IL18-driven activation, and the consequent CNS infiltration of CD8+ MAIT cells in MS, may cause reduced frequency in blood, helping to reconcile, at least in part, the conflicting results on the frequency of circulating MAIT cells in MS. A recent work showed indeed that MAIT cell subtype, smoking habit, and disease onset (primary progressive vs. relapsing–remitting) affect the number of circulating MAIT cells (22). Smokers with primary progressive MS showed low frequency of circulating MAIT cells, suggesting a tendency to reside in the inflamed organ, in apparent contrast to what was observed in most studies on patient with RRMS.

Concerning the effects of DMF on the variables under study, we found the expected decline of disease activity, which was in keeping with the initial pivotal trials (23–25). The parallel drop in the fraction of circulating CD161+CD8+ T cells is in accord with two previous works on the effects of DMF therapy in MS patients (22, 26). The action of DMF on all the proinflammatory T-cell subsets, including the CD161+ IL17-producing T cells, is mediated by a dose-dependent induction of apoptosis and decrease of proliferation (27). Other works, showing a decrease of proinflammatory MAIT cells after hematopoietic stem cell transplant or alemtuzumab for treatment-refractory forms (28, 29), support results obtained after DMF treatment, and indirectly confirm the pathogenic role of MAIT cells in MS.

No clear DMF effects were evident on IP changes, and the gastrointestinal side effects in our group of patients were relatively rare and apparently unrelated to IP changes. The meaning of this finding requires further studies (such as those based on novel multi-sugar assay for site-specific gastrointestinal permeability analysis) and suggests that the alterations of the gut barrier in MS are complex: the decreased disease activity at MRI and the reduction of the percentage of circulating MAIT cells during treatment with DMF seem to occur more frequently in patients with IP changes. These relationships raise the possibility that the gut barrier alteration may represent a predictor of pathophysiological transitions, besides its possible role in disease pathogenesis. Our study adds evidences to the potential role of mucosal immunity in MS pathogenesis, and yet suggests questions that remain unanswered. Among those are whether IP changes somehow drive demyelinating process [as seen in experimental models of MS; (9)] or simply contribute to the organ-specific immune dysfunction. Also, it is unclear through which mechanisms MAIT cells [or subsets of them; (22)] become activated and pathogenic at the CNS level in apparently sterile conditions. Answering these questions may provide new fruitful lines of attack against neuroinflammation, such as IP enhancers or stabilizers, already under scrutiny in gastro-intestinal conditions, as well as compounds coming from reworking the increasingly growing data coming from microbiota studies in experimental and human autoimmune diseases.

## Data Availability Statement

The raw data supporting the conclusions of this article will be made available by the authors, without undue reservation.

## Ethics Statement

The studies involving human participants were reviewed and approved by Azienda Ospedaliero-Universitaria Sant'Andrea, Università Sapienza. The patients/participants provided their written informed consent to participate in this study.

## Author Contributions

MSa, GR, and LB conceived the study. MB, GB, and MSi coordinated the project. FG, MC, and LL performed data analysis. CF carried out statistical analysis. All authors contributed to the project, writing of the manuscript, and approved its final version.

## Conflict of Interest

MSa receives research support and has received fees as speaker from Sanofi, Biogen, Roche, Novartis, Bayer Schering, and Merck Serono. LB received funds for research from TEVA, Baxter, Roche, and Merck Serono and speaker honoraria by Genzyme, Teva, Novartis, Roche, and Merck Serono. MB has received fees as speaker from Sanofi, Biogen, Roche, Merck Serono, and Novartis. The remaining authors declare that the research was conducted in the absence of any commercial or financial relationships that could be construed as a potential conflict of interest.

## Publisher's Note

All claims expressed in this article are solely those of the authors and do not necessarily represent those of their affiliated organizations, or those of the publisher, the editors and the reviewers. Any product that may be evaluated in this article, or claim that may be made by its manufacturer, is not guaranteed or endorsed by the publisher.
