# Supplementary material for: Intestinal Permeability and Circulating CD161+CCR6+CD8+T Cells in Patients With Relapsing–Remitting Multiple Sclerosis Treated With Dimethylfumarate
Source: Front Neurol. 2021 Aug 26;12:683398. doi: 10.3389/fneur.2021.683398 (PMC8426620; doi:10.3389/fneur.2021.683398)
Supplement: Supplementary file 1 [file Table_1.DOCX]

**Supplementary table**

| **ID** | **AGE**  **(ys-range)** | **DISEASE DURATION (ys)** | **CLINICAL ACTIVITY AT T°** | **EDSS** | **ACTIVITY AT MRI** | | | **INTESTINAL PERMEABILITY** | | |
| --- | --- | --- | --- | --- | --- | --- | --- | --- | --- | --- |
|  |  |  |  |  | T0 | T1 | T2 | T0 | T1 | T2 |
| **1** | 23-28 | 3 | S | 1 | S | S | S | a | n | a |
| **2** | 42-46 | 10 | S | 3,5 | A | S | S | a | a | n |
| **3** | 47-51 | 17 | S | 1 | S | S | S | a | a | n |
| **4** | 17-21 | 1 | R | 1 | A | S | S | a | a | a |
| **5** | 48-52 | 2 | S | 1 | A | S | S | n | a | a |
| **6** | 50-54 | 21 | S | 1,5 | A | S | S | a | a | a |
| **7** | 35-39 | 1 | S | 1,5 | A | A | S | a | a | a |
| **8** | 25-29 | 3 | R | 1,5 | S | S | A | a | a | n |
| **9** | 50-54 | 5 | S | 5 | S | S | S | a | a | a |
| **10** | 26-30 | 1 | S | 1 | A | S | S | n | a | n |
| **11** | 49-53 | 12 | S | 2,5 | S | S | S | a | n | a |
| **12** | 50-54 | 8 | S | 1,5 | A | S | S | a | a | n |
| **13** | 44-48 | 12 | S | 1 | S | S | S | a | a | n |
| **14** | 33-37 | 15 | S | 2,5 | A | A | S | a | a | n |
| **15** | 19-23 | 1 | S | 0 | A | S | S | a | n | a |
| **16** | 29-33 | 1 | R | 2 | S | A | S | n | a | a |
| **17** | 34-38 | 8 | S | 1 | S | S | S | a | n | n |
| **18** | 54-58 | 4 | S | 2 | S | A | S | n | n | a |
| **19** | 48-52 | 5 | S | 1 | S | A | S | n | n | a |
| **20** | 49-53 | 2 | S | 2 | S | S | S | n | n | a |
| **21** | 17-21 | 1 | S | 1,5 | A | A | S | a | n | a |
| **22** | 28-32 | 1 | R | 3 | A | S | S | n | a | a |
| **23** | 35-39 | 13 | S | 0 | A | S | S | n | a | a |
| **24** | 40-44 | 3 | S | 1 | A | S | S | n | n | n |
| **25** | 52-56 | 32 | S | 2 | A | S | S | a | a | a |

| **Abbreviations:** **EDSS**: expanded disability status scale; **MRI**: magnetic resonance imaging; **S**: stable; **R**=relapse **A**: active;  **n**: normal; **a**: altered. |  |  |  |  |  |  |
| --- | --- | --- | --- | --- | --- | --- |
